# 3D assessment of initial fracture displacement of tibial plateau fractures is predictive for risk on conversion to total knee arthroplasty at long-term follow-up

**DOI:** 10.1007/s00068-022-02139-y

**Published:** 2022-10-20

**Authors:** Nick Assink, Joep Kraeima, Anne M. L. Meesters, Mostafa El Moumni, Eelke Bosma, Robert J. Nijveldt, Sven H. van Helden, Jean-Paul P. M. de Vries, Max J. H. Witjes, Frank F. A. IJpma

**Affiliations:** 1 Department of Trauma Surgery, University of Groningen, University Medical Center HPC BA13, Hanzeplein 1, 9713 GZ Groningen, The Netherlands; 2 3D Lab, University of Groningen, University Medical Center Groningen, Groningen, The Netherlands; 3 Department of Trauma Surgery, Martini Hospital, Groningen, The Netherlands; 4 Department of Trauma Surgery, Isala Hospital, Zwolle, The Netherlands; 5 Department of Surgery, University Medical Center Groningen, Groningen, The Netherlands

**Keywords:** Tibial plateau fracture, 3D, Three-dimensional, Gap area, Displacement, Total knee arthroplasty

## Abstract

**Purpose:**

Currently used classification systems and measurement methods are insufficient to assess fracture displacement. In this study, a novel 3D measure for fracture displacement is introduced and associated with risk on conversion to total knee arthroplasty (TKA).

**Methods:**

A multicenter cross-sectional study was performed including 997 patients treated for a tibial plateau fracture between 2003 and 2018. All patients were contacted for follow-up and 534 (54%) responded. For all patients, the 3D gap area was determined in order to quantify the degree of initial fracture displacement. A cut-off value was determined using ROC curves. Multivariate analysis was performed to assess the association of 3D gap area with conversion to TKA. Subgroups with increasing levels of 3D gap area were identified, and Kaplan–Meier survival curves were plotted to assess survivorship of the knee free from conversion to TKA.

**Results:**

A total of 58 (11%) patients underwent conversation to TKA. An initial 3D gap area ≥ 550 mm^2^ was independently associated with conversion to TKA (HR 8.4; *p* = 0.001). Four prognostic groups with different ranges of the 3D gap area were identified: excellent (0–150 mm^2^), good (151–550 mm^2^), moderate (551–1000 mm^2^), and poor (> 1000 mm^2^). Native knee survival at 10-years follow-up was 96%, 95%, 76%, and 59%, respectively, in the excellent, good, moderate, and poor group.

**Conclusion:**

A novel 3D measurement method was developed to quantify initial fracture displacement of tibial plateau fractures. 3D fracture assessment adds to current classification methods, identifies patients at risk for conversion to TKA at follow-up, and could be used for patient counselling about prognosis.

**Level of evidence:**

Prognostic Level III.

## Introduction

Tibial plateau fractures are usually composed of complex fracture patterns consisting of multiple bone fragments with displacement in different directions. Achieving normal limb alignment and articular surface restoration is the main goal for surgical treatment. Achieving these goals is crucial to minimize the risk on posttraumatic osteoarthritis (OA) and the subsequent need for a total knee arthroplasty (TKA) [1]. However, adequate anatomical reduction cannot always be achieved due to comminution and severe initial fracture displacement. Meulenkamp et al. [2] recently reported that unsatisfactory reduction in fracture fragments occurs in up to 30% of the surgically treated tibial plateau fractures. Moreover, nearly anatomical reconstruction of severely displaced fractures may still result in early onset osteoarthritis [3, 4]. Altogether, irreversible initial damage to the articular surface (e.g., fracture displacement and comminution) affects to some extent the patient’s outcome.

Initial fracture displacement is decisive for the treatment strategy. Moreover, the initial damage to the joint mainly determines the prognosis [5]. The currently used classification systems and measurement methods are insufficient to assess fracture displacement [6]. The most frequently used classification systems (i.e., Schatzker, AO/OTA, Three-column [7–9]) describe fracture patterns instead of intra-articular incongruity. The degree of intra-articular fracture displacement is usually assessed by measuring the maximal gap and step-off on a single coronal, sagittal, or axial CT-slice. This method is known for its high inter- and intra-observer variability, tends to underestimate fracture displacement, and does not provide a full representation of the articular incongruity [10, 11]. Therefore, controversy remains within the literature regarding the association between the degree of initial intra-articular incongruity and the development of posttraumatic OA and functional recovery [12]. Consequently, patients with a tibial plateau fracture cannot properly be informed about their prognosis based on their fracture characteristics.

Recently, Assink et al. introduced a quantitative 3D CT (Q3DCT) method to quantify the intra-articular displacement in tibial plateau fractures [10]. This method showed superior reliability compared to 2DCT measurements and could be used as an addition to the current fracture classification systems [10]. This method involves the “3D gap area”, which quantifies the total surface area between all fracture fragments at the articular level. It includes all gaps and step-offs between all fracture fragments and represents a full quantification of the intra-articular incongruity. This current study aims to assess the association between the initial fracture displacement as measured in 3D and the risk of conversion to TKA at long-term follow-up.

## Methods

### Study design

A multicenter cross-sectional study was performed including all patients who have been treated for a tibial plateau fracture in three hospitals (one Level 1 and two Level 2 trauma centers) between 2003 and 2018. Patients were eligible for inclusion based upon the availability of a preoperative (diagnostic) CT scan of the injured knee with a slice thickness of ≤ 1 mm and a follow-up of at least 1 year. Patients with an isolated tibial eminence avulsion, a complicated fracture requiring amputation of the injured leg, age < 18 years, and those who had deceased or with an unknown address at the time of follow-up were excluded. Demographics were retrieved from the patients’ electronic records. For all patients, it was verified whether they were still alive according to the population registry. Patients were contacted by posted mail and asked whether they had conversion to a total knee prosthesis or not. Written informed consent was obtained from all participants. All available fracture types were included to avoid potential selection bias. The institutional review board of all centers approved the study procedures, and the research was performed in accordance with the relevant guidelines and regulations (research number: 201800411). This study is reported following Strengthening the Reporting of Observational Studies in Epidemiology (STROBE) reporting guideline [13].

### 2D imaging review

All CT scans at the time of the injury were reassessed by two independent observers in the axial, sagittal, and coronal planes to determine the fracture classification according to the Schatzker and the three-column classification systems [7, 9].

### 3D Fracture models

The data of the CT scan of the initial fracture of each patient was used to create a 3D fracture model. Mimics Medical software package (Version 21.0, Materialise, Leuven, Belgium) was used for this process. CT data (DICOM files, Digital Imaging and Communications in Medicine) were imported into the software after which a segmentation process was performed. A preset bone threshold (Hounsfield Unit ≥ 226) was used combined with the ‘region growing’ function in order to separate independent fragments. The segmentation was checked and if needed fragments were manually separated from adjacent fragments. Each fragment was assigned a different color and a smoothing filter was applied (factor 0.4).

### Introduction of the “3D gap area”

**Fig. 1:**
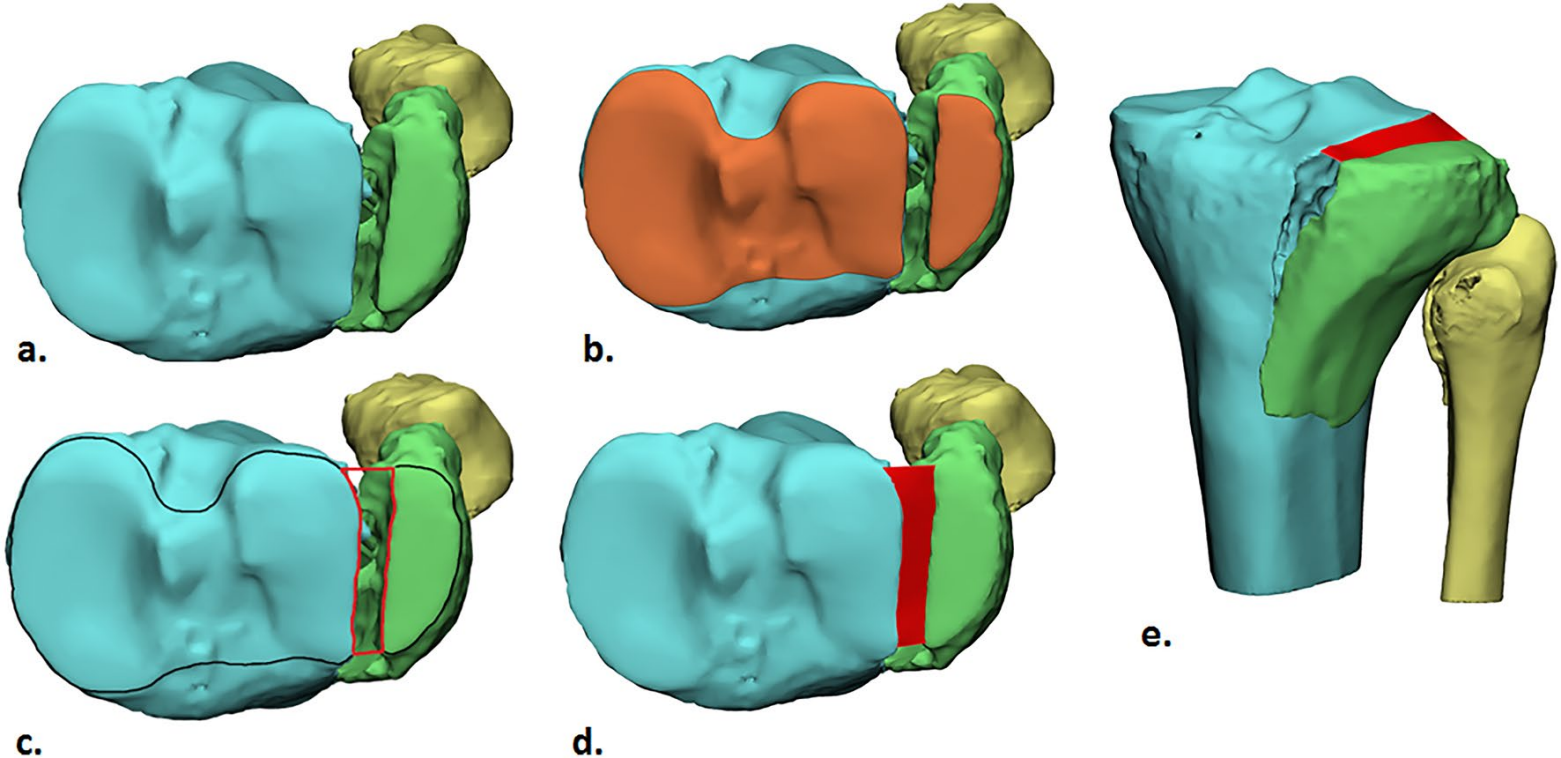
Method of measuring the 3D Gap area: **a** Cranial view of a lateral tibial plateau fracture (green); **b** Marking of the articular surface (orange); **c** Extracted contour of the articular surface (black line) from which the fracture lines are separated. The ends of the fracture lines are connected, resulting in an enclosed area (red lines); **d** The surface area between all fracture lines is measured resulting in a gap area (red surface) of 141 mm^2^ indicating an excellent prognosis; **e** Anterolateral view of the fracture with the measured gap area

**Fig. 2:**
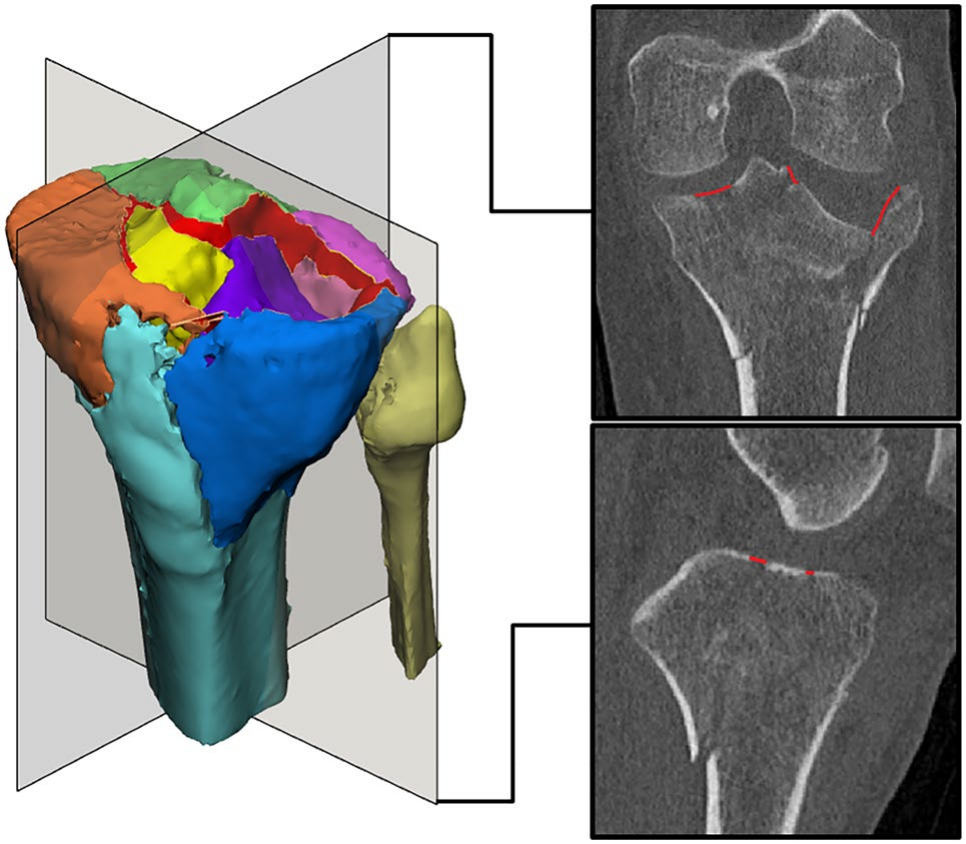
3D gap area (red surface) measurement on a 3D fracture model representing total fracture displacement between all fracture fragments at the articular level and the corresponding coronal and sagittal 2DCT slices

The 3D gap area was measured on the 3D fracture models (Fig. 1a) by using 3-matic Medical software (Version 13.0, Materialise, Leuven, Belgium). The 3D gap area is defined as the three-dimensional surface area between all fracture fragments and represents the total fracture displacement. It includes all gaps and step-offs between all fracture fragments and represents a full quantification of the intra-articular incongruity. In order to determine the 3D gap area, first, the articular surface was delineated on each fragment using the wave “brush mark” function (Fig. 1b). Secondly, the contours from the marked surface were extracted and trimmed with the “trim curve” function so that only the fracture lines at the articular surface level remained. After the fracture lines were separated, the ends of the fracture lines were connected resulting in an enclosed area (Fig. 1c). A 3D surface was constructed connecting all fracture lines by using the “surface construction” function in the 3-matic software (Fig. 1d). The surface, named 3D gap area, was measured in square millimeters (mm^2^). This area incorporates distances between fracture lines in all planes and is therefore considered a quantitative measure of the initial fracture displacement (Fig. 2).

### Study outcomes

This study intended to introduce and evaluate an innovative 3D measurement method—named 3D gap area—to assess initial fracture displacement of tibial plateau fractures. We hypothesize that a single 3DCT measurement for tibial plateau fractures will provide an observer independent CT-based analysis of the “initial damage to the joint” and could be a main factor that indicates whether a patient is at risk for a TKA during follow-up. The study did not aim to evaluate results (e.g., residual fracture displacement) after surgery, which is mostly based on radiographs and does not allow for advanced 3D measurements.

Our aim was to assess whether a detailed 3D measurement of initial fracture displacement is predictive for developing severe posttraumatic osteoarthritis with the subsequent need for a TKA. If the preoperative 3D gap area can be used to predict the risk of conversion to TKA during follow-up, this could have major implications for patient counselling about treatment options and expectations regarding the course of rehabilitation. To achieve our goal, we created 3D virtual fracture models based on the CT data of each patient and measured the “3D gap area” (three-dimensional surface area between all fracture fragments) on these 3D fracture models. Secondly, we contacted all patients asked whether they had conversion to total knee prosthesis (primary endpoint) at follow-up. To answer our research question, we assess the relationship between the 3D gap area (initial fracture displacement on the diagnostic CT-scan) and conversion to a total knee arthroplasty at long-term follow-up.

### Statistical analysis

Statistical analysis was performed using SPSS (version 23, IBM, Chicago, IL, US). Continuous variables were presented as mean and standard deviation (SD) for normally distributed data and median and interquartile range (IQR) if not normally distributed. A *p* value of less than 0.05 was considered statistically significant. Descriptive statistics were used to describe the study population. Mann–Whitney *U* test was performed to assess differences in baseline characteristics between responders and non-responders.

A receiver operating curve (ROC) was plotted to assess the association between initial fracture displacement as measured by the 3D gap area and conversion to a total knee arthroplasty at long-term follow-up. The area under the curve (AUC) was then determined to assess the diagnostic ability of the measurement. Critical cut-off for an increased risk on TKA was determined by using Youden’s J Statistics. Critical cut-off was defined as the value for which the combined sensitivity and specificity are the highest.

Kaplan–Meier curves of native knee survivorship were constructed for different subgroups with increasing 3D gap areas. The groups (excellent, good, moderate, and poor) were established based on a log relative hazard plot. Native knee survivorship curves were plotted and log-rank tests were performed to assess differences between groups with an excellent, good, moderate, or poor prognostic 3D gap area, respectively.

Cox regression analysis was performed to correct for other factors (age, sex, smoking, BMI, and inadequate articular reduction) which are potential confounders for the risk on osteoarthritis and conversion to a TKA [14–16]. Articular reduction (e.g., residual fracture displacement) was assessed on the first follow-up radiograph of each patient and considered adequate when both the maximum gap and step-off were ≤ 2 mm [17, 18].

### Funding statement

This research received no specific grant from any funding agency in the public, commercial or non-for-profit sectors.

## Results

### Patient demographics

Between 2003 and 2018, a total of 1220 patients were treated for a tibial plateau fracture in three hospitals of which 39 had and isolated tibial eminence avulsions (e.g., cruciate ligament injuries), four had an amputation, 50 were aged < 18 years, 112 had died at follow-up, and 18 had an unknown address, leaving 997 patients eligible for follow-up analysis. All patients were contacted by posted mail, from which 534 responded (response rate 54%) at a mean follow-up of 6.7 ± 3.6 years. Patient demographics are presented in Table 1. Non-response analysis demonstrated no differences between responders and non-responders in age (*p* = 0.067), gender (*p* = 0.478), and type of treatment (*p* = 0.96).

**Table 1:** Patient characteristics

Demographics	*N* = 534
Age (years)	53.1 (± 14.4)
Male	155 (29.0%)
BMI	26.3 (STD: 4.6)
Smoking	105 (19.6%)
Schatzker classification	
* Schatzker 1*	50 (9.4%)
* Schatzker 2*	200 (37.5%)
* Schatzker 3*	106 (19.9%)
* Schatzker 4*	57 (10.6%)
* Schatzker 5*	39 (7.3%)
* Schatzker 6*	82 (15.3%)
Three-column classification	
One column	174 (32.6%)
Two column	231 (43.3%)
Three column	129 (24.1%)
Operatively treated	372 (69.6%)
Conversion to TKA	58 (10.8%)
Follow-up (years)	6.7 (± 3.6)

### Initial fracture displacement related to TKA

**Fig. 3:**
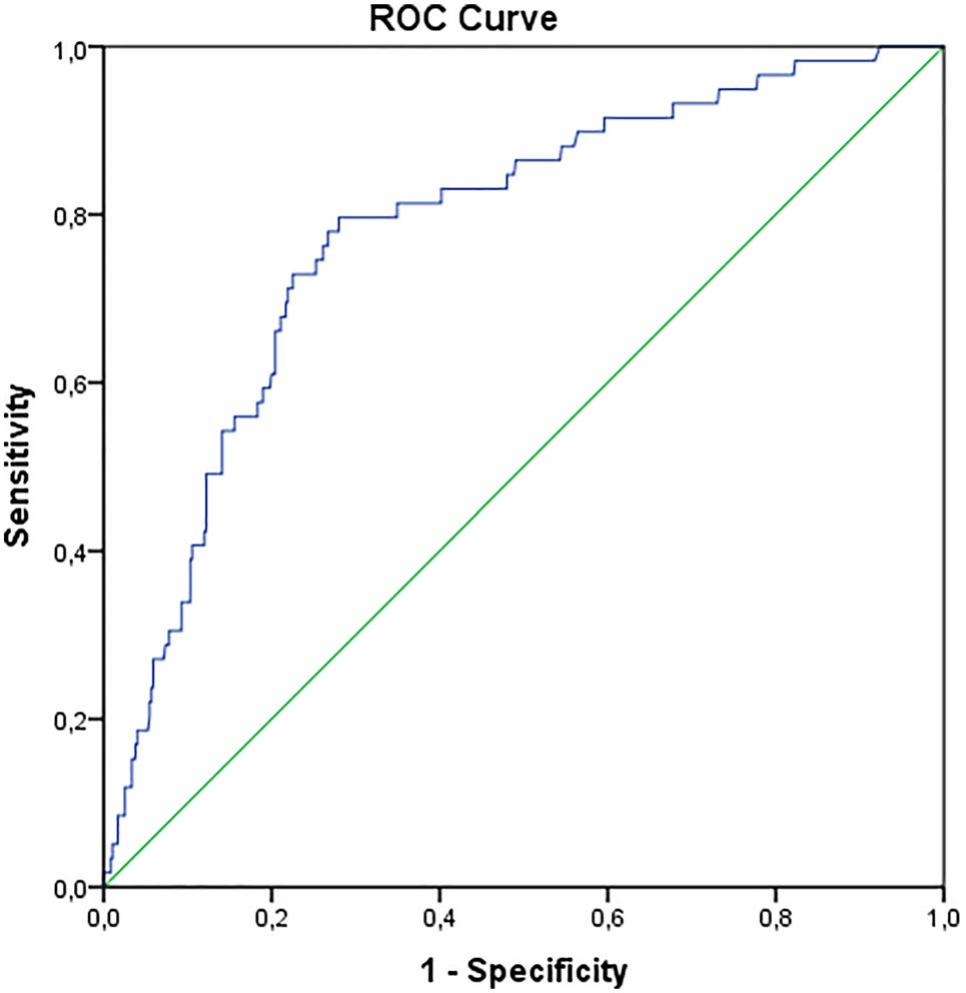
Receiver Operating Characteristic (ROC) curve demonstrating the 3D gap area associated with the conversion to total knee arthroplasty

Patients with conversion to a TKA compared to those who still had their native knee had significant more initial fracture displacement as measured by the 3D gap area (954.5 vs. 419.3 mm^2^, *p* < 0.001). The ROC curve showed an area under the curve of 0.78 for the 3D gap area associated with conversion to TKA (Fig. 3). The critical cut-off value, indicating the point that maximized sensitivity and specificity, was 550 mm^2^ (Table 2).

**Table 2:** 3D gap area cut-off values with their associated sensitivity, specificity, and Youden’s J derived from the ROC Curve analysis

3D gap area cut-off values (mm^2^)	Sensitivity (%)	Specificity (%)	Youden’s J
150	91.5	41.4	0.33
350	81.4	61.7	0.43
550^a^	76.3	73.9	0.50
750	59.3	81.2	0.41
950	49.2	86.3	0.36

^a^Critical cut-off point

### Native knee survival

**Fig. 4:**
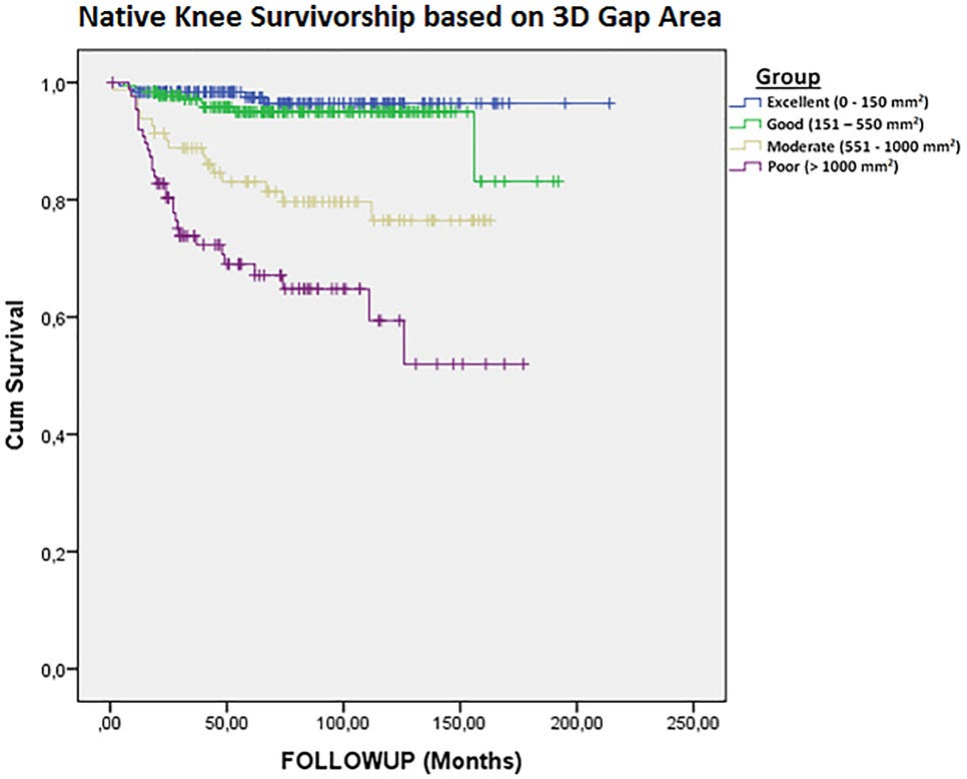
Kaplan–Meier curves of the native knee survival for fractures with respectively an excellent, good, moderate, or poor prognostic 3D gap area (log rank, *p* < 0.001)

**Fig. 5:**
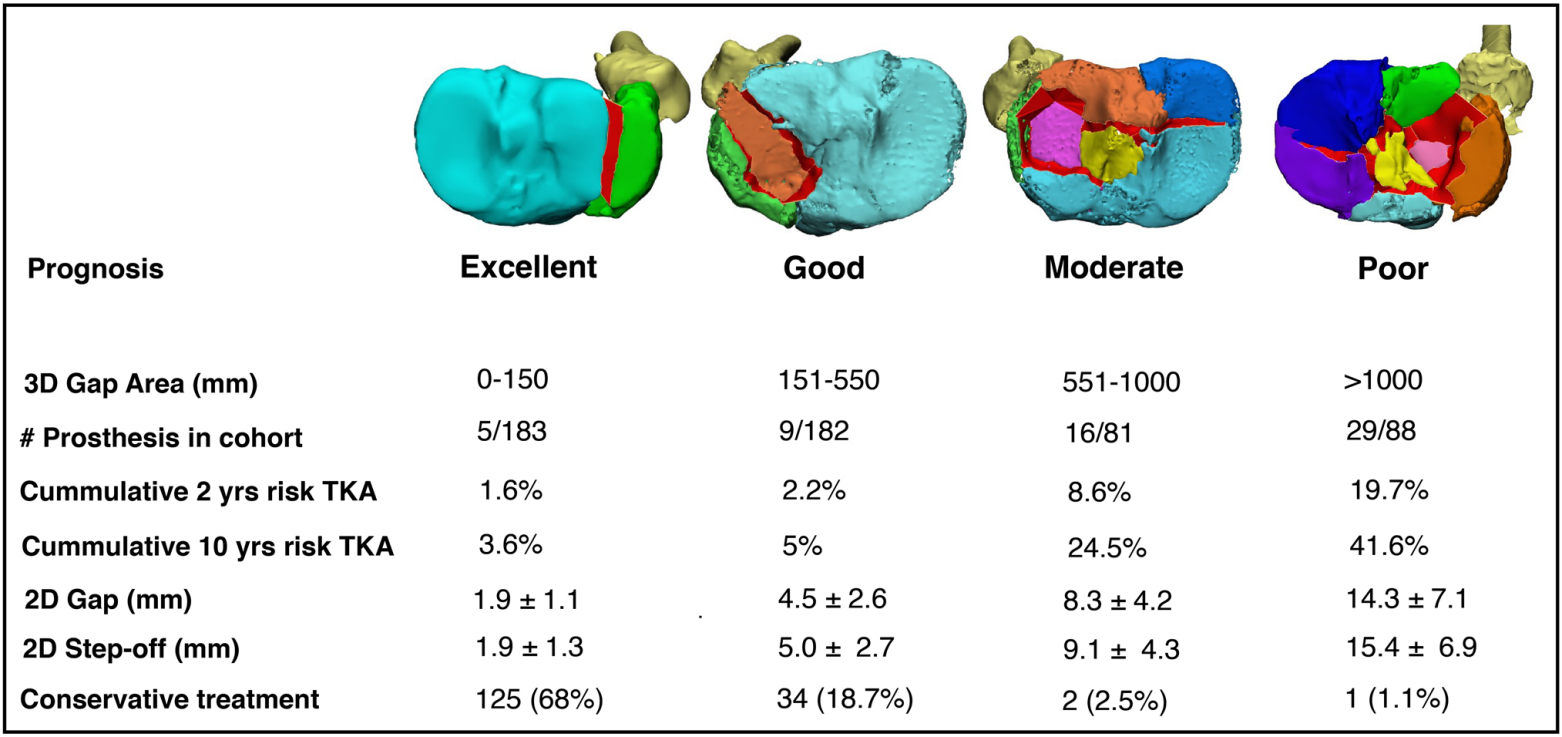
Four subgroups with increasing 3D gap area’s and their corresponding 2- and 10-year cumulative risk on conversion to TKA

From the plotted log relative hazard, two additional cut-off values for the 3D gap area (150 mm^2^ and 1000 mm^2^) were determined. Using these cut-off values for the 3D gap area, four prognostic groups were established: excellent (3D gap area 0–150 mm^2^), good (151–550 mm^2^), moderate (551–1000 mm^2^), and poor (> 1000 mm^2^). Kaplan–Meier survival curves (Fig. 4) show that at 2-years follow-up in the excellent prognostic group, 98.4% still have their native knee compared to 97.8% in the good group, 91.4% in the moderate group, and 80.3% in the poor group. At 10-years follow-up, the percentage of patients who still have their native knee was 96.4%, 95%, 75.5%, and 58.5%, respectively in the excellent, good, moderate, and poor group. Log-rank test showed a significant difference between the native knee survival distributions of the established prognostic four groups (*p* < 0.001). Cumulative risks for the conversion to a TKA for each of the prognostic subgroups are presented in Fig. 5.

Cox regression indicates that an increase in 3D gap area is independently associated with an increased risk on conversion to a TKA (HR 7.6, *p* < 0.001) (Table 3). Subgroup analysis showed that the hazard ratios increased alongside with the size of the 3D gap area. Compared to the excellent group (reference), the hazard ratios for conversion to TKA were respectively 1.7 (*p* = 0.34), 6.8 (*p* < 0.001), and 15.0 (*p* < 0.001) for the good, moderate, and poor prognostic group.

**Table 3:** Multivariate analysis, presenting unadjusted and adjusted hazard ratios for 3D gap area associated with conversion to TKA

Measure	Unadjusted hazard ratio (95% CI)	*p* value	Adjusted hazard ratio^a^ (95% CI)	*p* value
3D Gap Area ≥ 550 mm^2^	7.4 (4.1–13.5)	< 0.001	7.6 (4.1–14.4)	< 0.001
3D Gap Area subgroups				
Excellent (0–150 mm^2^; reference)	**–**	**–**	**–**	**–**
Good (151–550 mm^2^)	1.7 (0.6–5.1)	0.32	1.7 (0.6–5.1)	0.34
Moderate (551–1000 mm^2^)	6.8 (2.5–18.6)	< 0.001	6.8 (2.4–19.1)	< 0.001
Poor (> 1000 mm^2^)	14.3 (5.5–36.8)	< 0.001	15.0 (5.6–40.2)	< 0.001

^a^Adjusted for age, gender, smoking, BMI and inadequate reduction

## Discussion

The rationale for relating initial fracture displacement of tibial plateau fractures to risks on TKA at long-term follow-up is to inform the patients about their prognosis and guide treatment decisions shortly after the injury. Currently, objective measures for determining initial fracture displacement are lacking. This is the first study that introduced a quantitative 3D measure for initial fracture displacement and assessed the relationship between the degree of displacement in 3D and the risk of developing severe posttraumatic osteoarthritis and the subsequent need for a TKA. The 3D gap area is defined as a three-dimensional surface area between all fracture fragments and should be considered the next level of tibial plateau fracture assessment. Our findings demonstrate that the degree of initial fracture displacement is a strong predictor for conversion to a TKA. By defining clear cut-off values for the 3D gap area, a clear distinction could be made between groups with increasing risks on conversion to TKA at follow-up.

A limitation of this study is that our 3D analysis solely focused on preoperative fracture displacement. Ideally, the postoperative 3D gap area should be taken into account as well in order to assess and correct for the quality of the reduction. Unfortunately, this was not possible since postoperative CTs were not routinely performed in the participating centers. This is consistent with the current practice in many centers worldwide. Therefore, relationship between the 3D fracture reduction (i.e., residual fracture displacement) and clinical outcome could not be assessed. Regardless of the operation itself, clinical decision making based on initial fracture displacement is helpful for patient counselling regarding treatment options and prognosis in the early phase after the injury. As shown in this paper, there is an association between the severity of the injury and the risk of a TKA. Another limitation was the high variation in follow-up duration (12–214 months), which is inherent to a cross-sectional study design. Furthermore, since patients were contacted by posted mail some response bias can be expected within this study. Yet, no significant differences in age, gender and type of treatment were found between the responders and non-responders.

While the majority of studies, assessing the risk on posttraumatic osteoarthritis, focus on the quality of the surgical reduction, Marsh et al. [3] suggest that the most important factor in determining outcome should be considered damage to the articular surface caused by the injury. The initial damage may lead to some joint degeneration despite an accurate fracture reduction after surgery. In addition, Parkkinen et al. [5] concluded that the initial displacement of the fracture seems to have a role in the occurrence of posttraumatic OA. The results of our study are in line with previous findings and reconfirms that the degree of initial incongruity is indeed strongly associated with the risk of development of OA and eventually the need for conversion to TKA. However, this study adds to the results of previous studies that the degree of initial fracture displacement could be accurately quantified and stratified based on clinical follow-up data. Moreover, our study included the whole spectrum of initial fracture displacement ranging from minimally displaced nonoperatively treated fractures to severely displaced operatively treated fractures (Appendix 1 provides an overview of these varying cases). This study completely focusses on the relationship between initial fracture displacement and outcome regardless of type of treatment. Compared to the general population, patients with a tibial plateau fracture have a 5.3 times increased likelihood to undergo conversion to a TKA at ten-year follow-up regardless of the fracture severity [19]. This study, however, shows that this likelihood increases as the fracture severity increases. Patients in the good prognostic group are 1.8 times as likely to undergo conversion to a TKA compared to the excellent prognostic group. This likelihood increases up to 15.3 times for patients with major initial fracture displacement indicated as the poor prognostic group.

Our proposed 3D measurement method could be used as an addition to the current fracture classification methods in order to identify patients who are at risk for developing severe osteoarthritis resulting in conversion to TKA at follow-up. 3D fracture assessment could be used post-injury to fully inform the patient on the future risk on developing severe OA and the subsequent need for a TKA. Also, it could be used as a guideline for shared decision-making regarding treatment options taken into account these risks. This is especially true in the patients with a high risk on a TKA, since conversion to a TKA secondary to a tibial plateau fracture is associated with a higher rate of complications than TKA for primary osteoarthritis due to previous scars, bone loss and poor knee alignment [20–22]. Primary or early treatment with a TKA was found to be a suitable alternative in elderly patients with a complex fracture [20], and could be considered in these high-risk patients. Furthermore, it could be considered to minimize the surgical approach in poor prognostic patients to reduce complication rates (e.g., unnecessary fracture-related infections associated with multiple approaches in severely contused soft tissues) in the work-up to an early TKA. Besides the additional value of preoperative 3D fracture assessment, this measurement technique could also be applied on postoperative CT scans if needed in order to assess the quality of reduction.

Despite the potential benefits, the major limitation of performing the 3D fracture assessments is that it is labor-intensive. Depending on the fracture comminution, the segmentation and measurement process could take up to one hour. In the beginning, 3D fracture assessment of the initial displacement could therefore be reserved for selected cases in which there is a combination of substantial initial displacement and other known prognostic factors for conversion to TKA (i.e., increased age, smoking, BMI). Due to improvements in the segmentation software, the segmentation process is already semi-automatized. Yet, before widespread implementation in clinical practice, further automatization of the 3D measurements would be helpful.

In conclusion, we present an innovative 3D measurement method to quantify the degree of fracture displacement of tibial plateau fractures and correlated this to clinical outcome. Preoperative 3D fracture assessment could be used as an addition to the current fracture classification methods to identify patients who have a higher risk on developing progressive osteoarthritis and receiving a TKA at follow-up.

## Figures and Tables

**Fig. 6:**
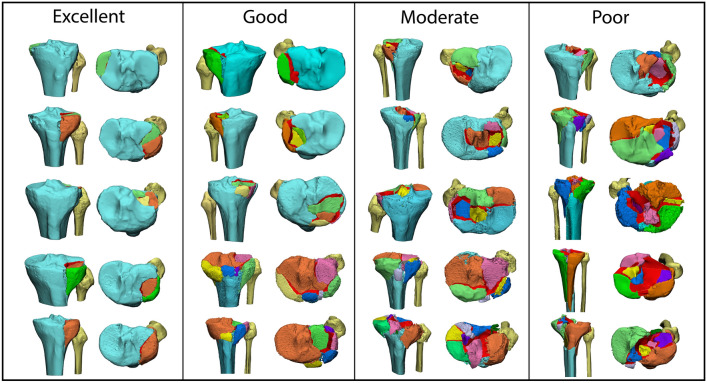
Overview of varying fractures which fall within the four identified subgroups

## Data Availability

Not applicable.

## Appendix 1

See Fig. 6.
